# A commentary on ‘Esketamine vs. placebo combined with erector Spinae plane block vs. intercostal nerve block on quality of recovery following thoracoscopic lung resection: a randomized controlled factorial trial’

**DOI:** 10.1097/JS9.0000000000002906

**Published:** 2025-07-04

**Authors:** Lei Shi, Shenghui Yu, Xiang Gao

**Affiliations:** Department of Anesthesiology, The Affiliated People’s Hospital of Ningbo University. Ningbo, Zhejiang, China

*Dear Editor,*

We read with great interest the recent article titled “Esketamine vs. placebo combined with erector spinae plane block vs. intercostal nerve block on quality of recovery following thoracoscopic lung resection: a randomized controlled factorial trial”^[1]^ published in the *International Journal of Surgery*. This study aims to evaluate the impact of erector spinae plane block (ESPB) versus intercostal nerve block (ICNB) in conjunction with subanesthetic esketamine versus placebo on postoperative recovery after thoracoscopic lung resection. According to a study, subanesthetic esketamine enhanced the quality of life (QoR) for patients undergoing thoracoscopic lung resection, and ICNB and ESPB can be used interchangeably as part of multimodal analgesia. However, a number of significant issues need to be addressed in future research.

First, the study employed a 2 × 2 factorial design to evaluate the impact of esketamine and regional nerve blocks (ESPB and ICNB) on postoperative recovery quality; however, it has several limitations. Firstly, the small sample size (25 participants per group) may limit statistical power, particularly in detecting interactions or differences in secondary outcomes. Secondly, the study did not include elderly patients (median age 57), which means the results may not be applicable to older populations. Additionally, while the study assumed independent effects of esketamine and regional blocks, it did not adequately explore potential synergistic effects between the two. The interaction effect was only briefly addressed through an interaction test (*P* = 0.215), without further analysis of the clinical significance of the combined treatment. Lastly, the study did not stratify the analysis by surgical type (such as lobectomy versus wedge resection), which could have obscured the impact of different surgical approaches on the outcomes.

Second, the study did not adjust for multiple comparisons of secondary outcomes, such as pain scores and opioid use, which heightened the risk of false positives. For example, the VAS score for cough in the PACU was significantly lower in the esketamine group compared to the placebo group (*P* = 0.009), but the uncorrected P value may have exaggerated its actual significance. Moreover, the clinical importance of secondary outcomes was not adequately discussed. For instance, the difference in QoR-15 scores at 48 hours and discharge (4.6 and 1.6 points, respectively) did not reach the predefined MCID (6.0 points), yet the study still considered it a positive outcome. The analysis of secondary outcomes lacked a pre-set hypothesis testing plan, potentially leading to selective reporting bias.

Third, the study did not provide detailed descriptions of the operational standardization processes for ESPB and ICNB, such as the diffusion range of local anesthetics or the objective assessment of block success (e.g., sensory block tests). ESPB relies on ultrasound guidance, whereas ICNB is visualized through thoracoscopy^[2]^. The operational differences between these two techniques may affect the comparability of block outcomes. Additionally, the study did not document cases of block failures or remedial measures, which could obscure technical heterogeneity^[3]^. Although the authors noted that all blocks were performed by experienced physicians, the lack of an evaluation of inter-operator consistency may introduce technical bias.

In summary, this study provides preliminary evidence for the use of esketamine and regional anesthesia in thoracoscopic surgery, but it has limitations in sample size, implementation of blinding, analysis of secondary outcomes, technical standardization, long-term follow-up, and external validity. Future studies can further validate these results by increasing the sample size, improving the blinding process, standardizing the assessment of block effects, extending the follow-up period, and adopting a multicenter design. All this was observed regarding the transparency in the reporting of Artificial Intelligence—the TITAN guideline^[4]^.

## Data Availability

No data was used in this Letter to the Editor.
